# Supplementary material for: Bronchoalveolar lavage affects thorax computed tomography of healthy and SARS-CoV-2 infected rhesus macaques (Macaca mulatta)
Source: PLoS One. 2021 Jul 9;16(7):e0252941. doi: 10.1371/journal.pone.0252941 (PMC8270458; doi:10.1371/journal.pone.0252941)
Supplement: S1 Table — (DOCX) [file pone.0252941.s001.docx]

S1 Table: Virus loads in swab samples of macaques following SARS-CoV-2 infection of the nose and throat..

| Viral Load Copies/ml | |  |  |  |  |  |  |  |
| --- | --- | --- | --- | --- | --- | --- | --- | --- |
| Nose | **day 0** | **day 1** | **day 2** | **day 3** | **day 4** | **day 6** | **day 7** | **day 8/9** |
|  |  |  |  |  |  |  |  |  |
| RM1 | neg | 1,33E+07 | 3,64E+08 | 6,43E+07 | 5,02E+05 | 8,27E+05 | 1,26E+06 | 5,44E+06 |
| RM2 | neg | 5,52E+05 | 9,00E+07 | 7,16E+05 | 3,19E+05 | 1,33E+06 | 1,11E+06 | 6,72E+06 |
| RM3 | neg | 1,05E+06 | 6,43E+07 | 3,81E+06 | 6,76E+05 | 5,49E+06 | 6,39E+07 | 5,35E+06 |
| RM4 | neg | 3,17E+03 | 9,00E+06 | neg | 1,99E+04 | 1,49E+07 | 8,57E+07 | 4,32E+07 |
| RM5 | neg | 4,83E+05 | 5,01E+06 | 2,68E+06 | 1,50E+05 | 6,60E+03 | 2,14E+03 | euthanized |
| RM6 | neg | 2,12E+05 | 1,99E+06 | 7,92E+04 | 9,57E+04 | 1,59E+05 | 3,26E+06 | euthanized |
| RM7 | neg | 2,71E+04 | 6,43E+02 | 3,43E+02 | neg | neg | neg | neg |
| RM8 | neg | 5,24E+04 | 6,00E+06 | 6,00E+07 | 4,62E+06 | 1,03E+05 | 1,47E+05 | neg |
| RM9 | neg | 1,63E+06 | 6,82E+04 | 3,06E+05 | 2,14E+03 | 2,91E+03 | 2,45E+04 | neg |
| RM10 | neg | 1,60E+04 | 3,86E+02 | 4,37E+03 | neg | neg | neg | neg |
| RM11 | neg | 1,58E+04 | neg | neg | neg | neg | neg | neg |
|  |  |  |  |  |  |  |  |  |
| Throat | **day 0** | **day 1** | **day 2** | **day 3** | **day 4** | **day 6** | **day 7** | **day 8/9** |
|  |  |  |  |  |  |  |  |  |
| RM1 | neg | 2,87E+06 | 4,16E+07 | 7,04E+05 | 8,10E+03 | 5,06E+03 | 2,29E+05 | 7,71E+04 |
| RM2 | neg | 2,44E+07 | 1,50E+07 | 2,61E+05 | neg | 7,14E+04 | neg | 3,36E+04 |
| RM3 | neg | 1,07E+09 | 2,49E+08 | 1,88E+06 | 3,98E+04 | 1,84E+04 | 2,23E+03 | 4,67E+03 |
| RM4 | neg | 4,71E+06 | 3,77E+08 | 2,96E+07 | 5,00E+05 | 2,39E+04 | 3,13E+04 | 1,83E+06 |
| RM5 | neg | 6,17E+06 | 4,97E+06 | 3,13E+05 | 2,44E+04 | 2,81E+04 | 5,39E+04 | euthanized |
| RM6 | neg | 1,07E+06 | 9,83E+05 | 1,15E+04 | 3,47E+03 | 3,00E+02 | 4,97E+04 | euthanized |
| RM7 | neg | 3,60E+04 | 2,70E+03 | 1,33E+03 | neg | neg | neg | neg |
| RM8 | neg | 3,56E+05 | 9,43E+05 | 2,13E+06 | 3,94E+05 | 2,96E+03 | 6,60E+03 | neg |
| RM9 | neg | 3,43E+05 | 5,67E+04 | 3,09E+04 | neg | 1,29E+02 | neg | neg |
| RM10 | neg | 3,17E+06 | 1,89E+07 | 1,47E+05 | 1,09E+05 | 2,23E+04 | neg | neg |
| RM11 | neg | 1,01E+05 | 3,86E+02 | neg | neg | neg | neg | neg |
