# Supplementary material for: Bronchoalveolar lavage affects thorax computed tomography of healthy and SARS-CoV-2 infected rhesus macaques (Macaca mulatta)
Source: PLoS One. 2021 Jul 9;16(7):e0252941. doi: 10.1371/journal.pone.0252941 (PMC8270458; doi:10.1371/journal.pone.0252941)
Supplement: S3 Table — (DOCX) [file pone.0252941.s003.docx]

S3 Table: overview of the CT scores of the left (le) and right (ri) lung of the SARS-CoV-2 infected rhesus macaques (RM)

|  | CT score pre | | CT score 24h post | |
| --- | --- | --- | --- | --- |
| RM | **le** | **ri** | **le** | **ri** |
| 1 | 0 | 2 | 0 | 1.5 |
| 2 | 1 | 0 | 1 | 0 |
| 3 | 5 | 0 | 10 | 0 |
| 4 | 6 | 4 | 11 | 6 |
| 5 | 3 | 5 | 6 | 5 |
| 6 | 3 | 1 | 9 | 1 |
| 7 | 0 | 0 | 4 | 0 |
| 8 | 1.5 | 0 | 1 | 0 |
| 9 | 2 | 0 | 4 | 0 |
| 10 | 0 | 5 | 0 | 7 |
| 11 | 1 | 6 | 3 | 2 |
