# Supplementary material for: Bronchoalveolar lavage affects thorax computed tomography of healthy and SARS-CoV-2 infected rhesus macaques (Macaca mulatta)
Source: PLoS One. 2021 Jul 9;16(7):e0252941. doi: 10.1371/journal.pone.0252941 (PMC8270458; doi:10.1371/journal.pone.0252941)
Supplement: S1 Fig — (DOCX) [file pone.0252941.s004.docx]

S1 Fig: histograms representing the relative lung density pattern of the left and right lung of RM1 of the non-infected group. LD= -1000/-600 HU, healthy lung, MD= -600/-400 HU, HD= -400/0 HU, non-healthy lung.
